# Macrophage migration inhibitory factor contributes to immunopathogenesis during *Plasmodium yoelii* 17XL infection

**DOI:** 10.3389/fcimb.2022.968422

**Published:** 2022-08-24

**Authors:** Víctor H. Salazar-Castañón, Imelda Juárez-Avelar, Martha Legorreta-Herrera, Miriam Rodriguez-Sosa

**Affiliations:** ^1^ Laboratorio de Inmunología Molecular, Unidad de Investigación Química Computacional, Síntesis y Farmacología en Moléculas de Interés Biológico, División de Estudios de Posgrado e Investigación, Facultad de Estudios Superiores Zaragoza, Universidad Nacional Autónoma de México (UNAM), Ciudad de México, Mexico; ^2^ Laboratorio de Inmunidad Innata, Unidad de Investigación en Biomedicina, Facultad de Estudios Superiores Iztacala, UNAM, Estado de México, Mexico

**Keywords:** *P. yoelii* 17XL, host-pathogen interaction, parasite, MIF, malaria, proinflammatory cytokines, pathogenesis, immune response

## Abstract

Macrophage migration inhibitory factor (MIF) is a cytokine recognized regulator of the inflammatory immune response associated with several immune cells that produce inflammatory cytokines such as IL-1β, IL-6, IL-12, IL-18, and TNF-α. This study aimed to understand the effect of MIF on the immune response and pathogenesis during *Plasmodium* infection. Wild-type (Wt) and MIF knockout (*Mif* ^-/-^) mice were intravenously infected with 1×10^3^ *Plasmodium yoelii* (*Py*) 17XL-parasitized red blood cells. Our data showed that *Py*17XL-infected Wt mice died 11 days postinfection, while *Mif* ^-/-^ mice showed reduced parasitemia and an increase in their survival at day 11 up to 58%, importantly they succumb up to day 21 postinfection. The increased survival rate in *Mif* ^-/-^ mice was associated with less severe cachexia and anemia as a result of a mixed Th1/Th2 cytokine profile, high levels of IL-12, IL-17/IL-4, and IL-10 in serum; and high levels of IL-4 and IL-10, and low levels of IFN-γ in spleen cells compared to *Py*17XL infected Wt mice. Moreover, macrophages (Mφs) from *Mif* ^-/-^ mice exhibited higher concentrations of IL-10 and IL-12 and reduced levels of TNF-α and nitric oxide (NO) compared to *Py*17XL-infected Wt mice. These results demonstrate that MIF has an important role in regulating the immune response associated with host pathogenesis and lethality, which is relevant to consider in preventing/reducing complications in *Plasmodium* infections.

## Introduction

Malaria is one of the main health problems globally, causing more than 241 million clinical cases and resulting in 627,000 deaths in 2020, in accordance with the last report by the World Health Organization (WHO, 2021). Death is mainly due to complications of *Plasmodium* infection, such as severe anemia and cerebral malaria. Although dysregulation of host proinflammatory cytokines plays a crucial role in pathogenesis and lethality, the fine mechanism of this dysregulation is not fully understood (Clark et al., 2008; Bakir et al., 2011).

Resistance to *Plasmodium* infections has been associated with an early inflammatory Th1-type immune response characterized by IL-12, TNF-α, and IFN-γ production (Langhorne et al., 2004), followed by antiinflammatory Th2 response characterized by IL-4 and IL-10, which is required to control chronic parasitemia and avoid pathologic damage due to exacerbated inflammatory responses (Schofield and Grau, 2005). Deficient or excessive inflammatory cytokine responses may alter the course of infection and contribute to pathogenesis.

Macrophage migration inhibitory factor (MIF) is a pleiotropic cytokine involved in immunomodulatory functions in the immune response. Mφs and T cells produce substantial quantities of MIF within cytoplasmic vesicles released upon cell stimulation (Kang and Bucala, 2019). MIF is required for NOD-, LRR- and pyrin domain-containing protein 3 (NLRP3) inflammasome activation and plays a pivotal role in the inflammatory process by affecting several immune cells, which produce inflammatory cytokines such as IL-1β, IL-6, IL-12, IL-18, and TNF-α (Lang et al., 2018). In addition, MIF regulates glucocorticoid immunosuppressive effects (Leng et al., 2009) and supports and enhances macrophage proinflammatory functions by preventing p53-mediated apoptosis (Roger et al., 2001). These findings explain how MIF plays an important role in the pathogenesis of several inflammatory disorders, such as sepsis (Pohl et al., 2017), inflammatory bowel disease (IBD) (Nishihira and Mitsuyama, 2009), allergic (Xie et al., 2021) rheumatoid arthritis and lupus (Bilsborrow et al., 2019). Moreover, MIF is also involved in the protection of several parasitic infections (Rosado Jde and Rodriguez-Sosa, 2011; Filbey et al., 2019), such as *Taenia crassiceps* (Rodriguez-Sosa et al., 2003), *Toxoplasma gondii* (Terrazas et al., 2010), and *Trypanosoma cruzi* (Fu et al., 2012a).

Numerous studies have shown that *Plasmodium* infection induces high levels of MIF in humans or mice and have suggested that the proinflammatory response promoted by MIF might affect the outcomes of *Plasmodium* infection; however, there is no consensus on the role of MIF in severe malaria. Some studies report that *Plasmodium* infection induces high levels of MIF in humans or mice and suggest that the proinflammatory response promoted by MIF is responsible for the pathology, severe malaria, and fatal outcomes (Jain et al., 2009; Baeza Garcia et al., 2021). Other studies have shown that low MIF production increases the severity of the infection (Awandare et al., 2006; Awandare et al., 2007a; Awandare et al., 2007b; De Mast et al., 2008). Nonetheless, this discrepancy could be solved if these observations were confirmed and extended to different *Plasmodium* strains, indicating whether the role of MIF differs according to the timing and the magnitude of the production of proinflammatory cytokines promoted by MIF.

To contribute to the understanding of the role of MIF in the immune response and pathogenesis during *Plasmodium* infection, we developed an MIF-deficient mouse malaria model using *Mif* ^-/-^ mice infected with *Plasmodium yoelii* (*Py*)17XL. This is a strain of *Plasmodium* adapted in the laboratory to study immune mechanisms, identification of vaccines, and severe malaria and cerebral malaria caused by *Plasmodium falciparum* (Lamb et al., 2006; Salazar-Castanon et al., 2014). Infection with *Py*17XL results in rapid parasite growth, body weight loss, splenomegaly, severe anemia, lesions in the brain, liver, kidney, lung, and death 7 to 11 days postinfection in most mouse genetic backgrounds (Fu et al., 2012b). This model, which focuses on the role of MIF, demonstrates that MIF plays a fundamental role in the regulation of the immune response associated with the pathogenesis and host lethality of malaria.

## Materials and methods

### Mice

Six- to eight-week-old female BALB/cAnN mice were purchased from Harlan Laboratories (ENVIGO, México), and *Mif ^-/-^* mice were developed as described previously (Bozza et al., 1999). Briefly, the MIF gene was disrupted by replacing part of exons 2 and 3 with a *neo* ^r^ cassette. The targeting vector was electroporated into J1 ES cells, and G418-FIAU-resistant colonies were isolated. Targeted ES cells were used to generate chimeric animals by injection into C57BL/6 blastocysts. Highly chimeric animals transmitted the mutated allele through the germline, and homozygous mice were generated by intercrossing heterozygous mice as previously described (Bouabe and Okkenhaug, 2013). Finally, mice were backcrossed for more than 10 generations to a BALB/c genetic background. Mice were maintained in a pathogen-free environment at the Facultad de Estudios Superiores Iztacala, UNAM, México.

### Parasite and infection

*Plasmodium yoelii* 17XL was kindly donated by Dr. W. Jarra National Institute for Medical Research, Mill Hill, London, UK. The parasites were cryopreserved in liquid nitrogen. Infections were performed by intravenous injection with 1×10^3^ *Py*17XL-parasitized red blood cells (pRBCs). Three to six mice in each group were infected with *Py*17XL. Noninfected *Mif ^-/-^* and Wt mice were used as controls. To monitor the survival rates, two experiments were performed. For cytokine measurements and spleen analyses, mice were euthanized under a CO_2_/O_2_ atmosphere on the 7^th^ day after *Py17*XL infection

### MIF quantification

The MIF protein concentration was measured in the serum. Blood samples from the control and experimental groups at 5 and 7 days postinfection with *Py*17XL were obtained from the tail vein, and the serum was collected by centrifugation at 700 × g for 10 min in a Prism R microcentrifuge (Labnet International, Woodbridge, NJ, USA) and stored at -20°C until use. Fifty microliters of serum was used for MIF quantification using the mouse MIF DuoSet Sandwich enzyme-linked immunosorbent assay (ELISA) kit (R&D Systems, Minneapolis, MN, USA) in accordance with the manufacturer’s instructions. The samples were read, and the optical density (OD) was measured using an Epoch microplate spectrophotometer scanner at 405 nm (BioTek, Winooski, VT, USA). A representative ELISA curve for converting OD is shown in **Figure S3**.

### Severity of disease

Disease was assessed by survival, parasitemia, weight loss (cachexia) and decreased hemoglobin concentration (as an indicator of anemia). Following *Py*17XL infection, mice were monitored daily to document mortality and body weight loss using an electronic scale (Citizen CX series, Mumbai, India).

### Parasitemia

Parasitemia counts were conducted under oil using a Zeiss Standard 20 microscope (Carl Zeiss Ltd., Welwyn Garden City). When the number of parasitized red blood cells exceeded 0.5%, we counted 200 red blood cells. Lower levels of parasitemia were assessed by counting the parasitized erythrocytes present in 50 fields. The course of infection in each group is presented as the geometric mean of the parasitemia percentage.

### Quantification of hemoglobin concentration

The hemoglobin (Hb) concentration was measured by a colorimetric method by diluting 2 µL of blood in 498 µL of *Drabkin’s* reagent (sodium bicarbonate, potassium ferricyanide, and potassium cyanide; Sigma–Aldrich, St. Louis, MO, USA). The absorption at 540 nm was measured using an Epoch microplate spectrophotometer (Biotek, Winooski USA). The Hb concentration was calculated using a commercial standard curve.

### Spleen index

On day 7 after *Py*17XL infection, spleen and body weights were assessed. To calculate the splenic index, the spleen weight value was divided by the weight of the mouse.

### *Plasmodium yoelii* 17XL antigen (*Py*Ag) preparation

*Py*Ag was obtained as described elsewhere (Lucas et al., 1993). Briefly, mice with 20-30% parasitemia were euthanized, and blood was collected by cardiac puncture and pooled in heparinized tubes containing PBS, pH 7.2. The blood was passed through a column of cellulose CF11 to eliminate white blood cells. The red blood cells were washed two times with PBS by centrifugation for 5 min at 750 g. Red blood cells were lysed with 0.06% saponin (Merck, Darmstadt, Germany) in PBS by incubating samples at room temperature for 10 min. After lysis of red blood cells, the released parasites were washed two times with PBS by centrifugation for 15 min at 18,000 g, and the resulting pellet was lysed in lysis solution containing 1% Triton X-100, 100 mM Tris-HCl pH 8, and 5 mM EDTA. Nonsoluble material was eliminated by centrifugation at 20,000 g for 30 min. The protein concentration in the soluble antigenic material was quantified with protein Bio-Rad reagent (Bio-Rad, Richmond, CA, USA). Red blood cell extracts from noninfected mice were prepared and used as controls.

### Macrophage culture

Peritoneal exudate cells (PECs) were obtained from the peritoneal cavity 7 days after infection under sterile conditions using 10 mL of ice-cold Hank´s balanced salt solution (Microlab, Mexico). Following two washes with Hank’s balanced saline solution, red blood cells were lysed by resuspending the cells in Boyle’s solution (0.17 M Tris and 0.16 M ammonium chloride, all from Sigma Aldrich). The viable cells were counted using the trypan blue exclusion method (routinely exceeding 95%) with a Neubauer hemocytometer (Sigma–Aldrich). PECs were adjusted to 5x10^6^ cells/mL in DMEM supplemented with 10% fetal calf serum, 100 U of penicillin/streptomycin, and 2 mM glutamine (all from Gibco-BRL, Grand Island, NY) and cultured in 24-well plates (Costar, Cambridge, MA, USA). After 2 h of incubation at 37°C and 5% CO_2_, nonadherent cells were removed by washing with warm DMEM. Adherent cells (Mφ) were removed from the plate by washing with 5 mM EDTA in warm PBS and then adjusted to 1×10^6^ cells/mL. These constituted >85% of the Mφ according to flow cytometry analysis. Briefly, Mφs were incubated with 10 μg/mL anti-CD16/32 in stain buffer (1X PBS, 2% FBS, 1% NaN_3_) for 15 min, followed by staining for 20 min with the Mφ marker antibody (Ab) APC-conjugated anti-F4/80, as well as the isotype control Ab. Mφs were washed with FACS buffer and fixed in 0.8% paraformaldehyde before acquisition and analysis (Attune NxT Applied Biosystems, Waltman, MA, USA, all antibodies were purchased from Biolegend). One million Mφs were plated into 12-well plates (Costar) and left untreated or stimulated with total *Py*Ag (25 μg/mL). Mφs were incubated for 48 h at 37°C and 5% CO2. Supernatants were collected and stored at -20°C until used for cytokine quantification (TNF-α, IL-12 and IL-10).

### Spleen-cell proliferation

Spleens were aseptically removed from infected and control mice under sterile conditions and the spleen cells were disaggregated in a nylon mesh with cold PBS and resuspended in DMEM supplemented with 10% fetal bovine serum, 100 units of penicillin/streptomycin, and 2 mM glutamine (all from Gibco). Splenocytes were adjusted to a density of 5×10^6^ cells/mL in the same medium. One hundred microliters of the cell suspension were seeded in 96-well flat-bottom culture plates (Costar) and stimulated with either concanavalin-A (2 µg/mL) (Con-A; Sigma–Aldrich) or *Py*Ag (25 µg/mL). The plates were incubated at 37°C and 5% CO_2_ for 72 h or 5 days with Con-A or *Py*Ag, respectively. Eighteen hours before culture termination, 0.5 µCi of tritiated thymidine (Methil-^3^H)-20Ci 1 mCi/mL (37 MBq/mL) NET027E 20 Ci/mmol, METRIX Lab, CD MX) was added to each well. After further incubation for 18 h, the cells were harvested using a 96-well harvester (Tomtec, Hamden, USA) and counted using a microplate counter (Trilux, Hamden, USA). The results are presented as counts per minute (CPM). Supernatants were collected and stored at -20°C until they were used in cytokine assays to determine the IFN-γ, IL-4 and IL-10 levels.

### Quantification of cytokines

Spleen cell and Mφ culture supernatants described above and serum samples from the control and experimental groups at 7 days postinfection with *Py*17XL were collected and stored at -20°C until use. The serum levels of proinflammatory cytokines (IL-12, IFN-γ and TNF-α) and anti-inflammatory cytokines (IL-4 and IL-10) were analysed using commercially available enzyme-linked immunosorbent assay (ELISA, all from Peprotech, New Jersey, USA). The samples from spleen and Mφ cultures were diluted 1:2 in PBS, whereas the serum samples were diluted 1:2 or 1:4 in PBS as necessary. The samples were tested according to the manufacturer’s instructions (Peprotech). The optical density (OD) was measured using an Epoch microplate spectrophotometer reader (Biotek) at 405 nm. A representative curve for each ELISA cytokine for OD conversion is shown in **Figure S3**.

### Statistical analysis

Normality was tested by analysing the Fisher-Pearson standardized third moment coefficient, and homogeneity of variance was analysed using Levene’s test. When heterogeneity of variance was detected, Welch´s ANOVA was used. Parasitemia, body mass and hemoglobin concentration were analysed using ANOVA for repeated measurements. The splenic index, differences in cytokine levels and proliferation of spleen cells were analysed using ANOVA, and Fisher’s LSD method was used for multiple comparisons. The survival data were analysed using the log-rank Mantel Cox test. Values of p < 0.05 were considered statistically significant.

## Results

### *Plasmodium yoelii* 17XL infection increases MIF levels in serum

Major complications from *Plasmodium* infection have been associated with strong innate immune activation and pronounced proinflammatory cytokine production in response to *Plasmodium* parasites and/or their released metabolites. To confirm that *Py*17XL infection induces the secretion of MIF and validate the lack of MIF in *Mif ^-/-^* mice, we evaluated the serum concentration of MIF in *Py*17XL-infected *Mif ^-/-^* and Wt mice at 5 and 7 days postinfection.

**Figure 1:**
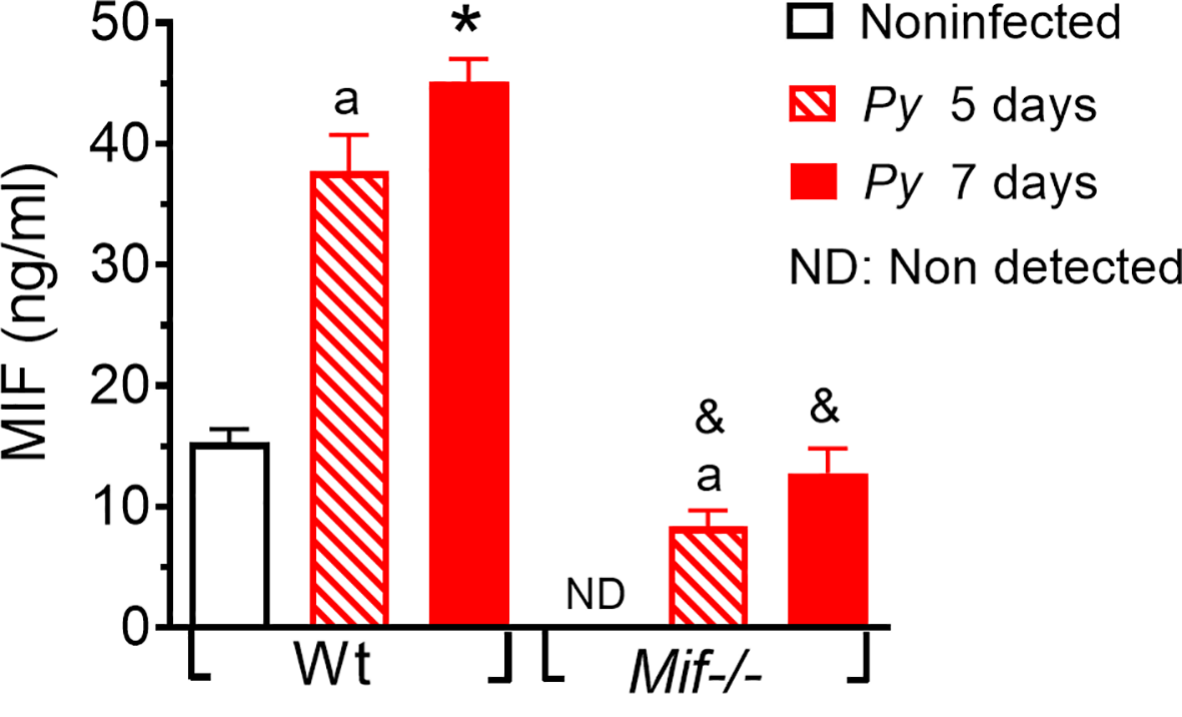
*Plasmodium yoelii* 17XL infection increases MIF levels in serum. Wild-type and *Mif ^-/-^* mice were intravenously infected with 1×10^3^ *Py*17XL-parasitized erythrocytes. All experimental groups were bled at 5 and 7 days postinfection, and MIF protein levels were measured in serum. Data are expressed as the mean ± SEM and are representative of 3 independent experiments with at least 3 to 5 mice per group. Values of p < 0.05 were considered statistically significant, (a) compared with noninfected Wt mice, (*) compared with all experimental groups, (&) compared with *Py*17XL at 5 days postinfection.

As shown in **Figure 1**, Wt mice had basal levels of MIF but after *Py*17XL infection it increased significantly on days 5 and 7 postinfection. These data suggest that MIF may be involved in the inflammatory response to *Py*17XL infection. Basal levels of MIF were not detected in uninfected*Mif ^-/-^* mice; however, MIF was detected in *Mif ^-/-^* mice on days 5 and 7 postinfection, although the levels achieved were lower than those in uninfected Wt mice. It is likely that serum concentrations of MIF detected in the infected *Mif ^-/-^* could be released from the *Plasmodium* parasite rather than from the host cells, as previously reported (Thorat et al., 2010).

### MIF deficiency delays the host mortality induced by *Py*17XL infection

Since MIF was expressed during malaria infection, we examined whether MIF affects the course of *Py*17XL infection. Wt and *Mif ^-/-^* mice were infected with *Py*17XL-parasitized erythrocytes, and parasitemia and survival rates were assessed in all groups.

**Figure 2:**
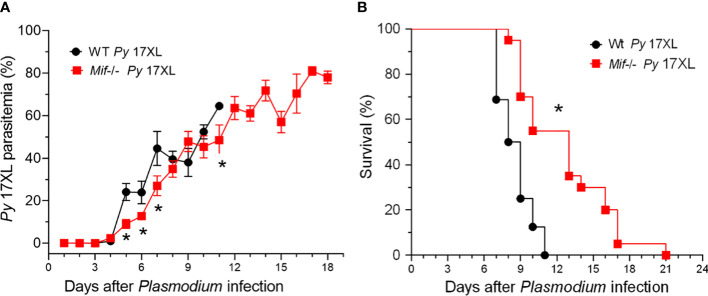
MIF deficiency delays the host mortality induced by *Plasmodium yoelii* 17XL infection. Wild-type and *Mif ^-/-^* mice were intravenously infected with 1×10^3^ *Py*17XL-parasitized erythrocytes. **(A)** Parasitemia is expressed as the mean % of *Py*-parasitized erythrocytes in each group. **(B)** Survival rate for each experimental group. Data are expressed as the mean *±* SEM and are representative of 3 independent experiments with 3 to 5 mice per group. Values of *p < 0.05 were considered statistically significant relative to the *Py*17XL-infected Wt mice.

*Mif* ^-/-^ mice infected with *Py*17XL showed reduced parasitemia at 5, 6, 7 and 11 days after infection compared to Wt mice. However, on day 8 they reached parasitemia similar to that of Wt mice (**Figure 2A**). Importantly, infected *Mif ^-/-^* mice showed a significant increase in survival rate (**Figure 2B**). While Wt mice succumbed on day 11, *Mif ^-/-^* mice succumbed until day 21 postinfection (*p<0.0001). Moreover, at day 11, when all Wt mice succumbed (median=8 days 95% CI), 58% of infected *Mif ^-/-^* mice were alive (median=12 days 95% CI).

These findings demonstrate that MIF is involved in susceptibility, which leads to early mortality, independent of *Py*17XL replication.

### MIF deficiency delayed the development of cachexia and reduced the early loss of hemoglobin after *Py*17XL infection

To understand how MIF deficiency increases the survival of mice infected with *Py*17XL, cachexia associated with weight and anemia were examined, both parameters related to the pathology and severity of *Plasmodium* infection. Body weight loss and hemoglobin concentration in infected *Mif ^-/-^* and Wt mice were monitored every day.

**Figure 3:**
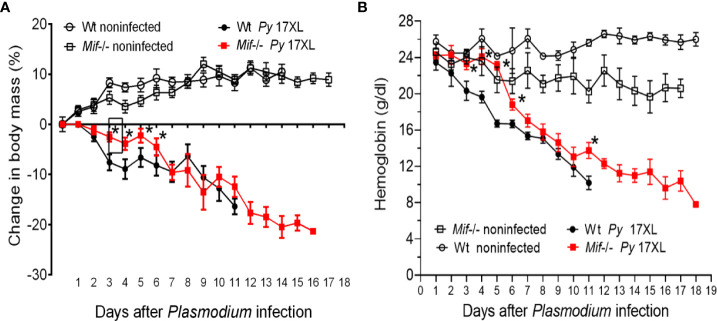
MIF delayed the development of cachexia and reduced the early loss of hemoglobin after *Plasmodium yoelii* 17XL infection. Wild-type and *Mif ^-/-^* mice were intravenously infected with 1×10^3^ *Py*17XL-parasitized erythrocytes. Non infected Wt mice were used as controls. **(A)** Body mass change and percentage weight change were calculated using the weight of each animal at day 0. **(B)** Hemoglobin concentration. Data are expressed as the mean ± SEM and are representative of 2 independent experiments with at least 3 to 5 mice per group. Values of p < 0.05 were considered statistically significant (*) compared to *Py*17XL-infected Wt mice.

Infected Wt mice gradually lost weight from day 3 to day 11, losing approximately 16% of their weight. In the same period, from days 3 to 11, infected *Mif ^-/-^* mice lost approximately 12% of their weight (**Figure 3A**). Importantly, between days 3 and 5 postinfection, *Mif ^-/-^* mice lost less weight and did not exhibit low hemoglobin levels compared to infected Wt mice (**Figures 3A, B**, respectively). *Mif ^-/-^* mice continued to lose weight until day 21, achieving a 21% weight loss, but hemoglobin loss was lower than that in Wt mice (**Figures 3A, B**, respectively).

Taken together, these data indicate that MIF deficiency delayed the development of cachexia and reduced early anemia and suggest that mortality does not seem to be fully related to weight loss.

### MIF deficiency reduces the development of splenomegaly after *Py*17XL infection

The spleen is the organ where the malaria parasite is eliminated by the immune response, and it is involved in resistance to severe anemia; therefore, excessive inflammatory responses triggered by *Plasmodium* infection promote splenomegaly (Leoni et al., 2015). This condition correlates with the outcome of the disease and can be used as a clinical marker to estimate the pathogenesis of malaria (Huang et al., 2016).

**Figure 4:**
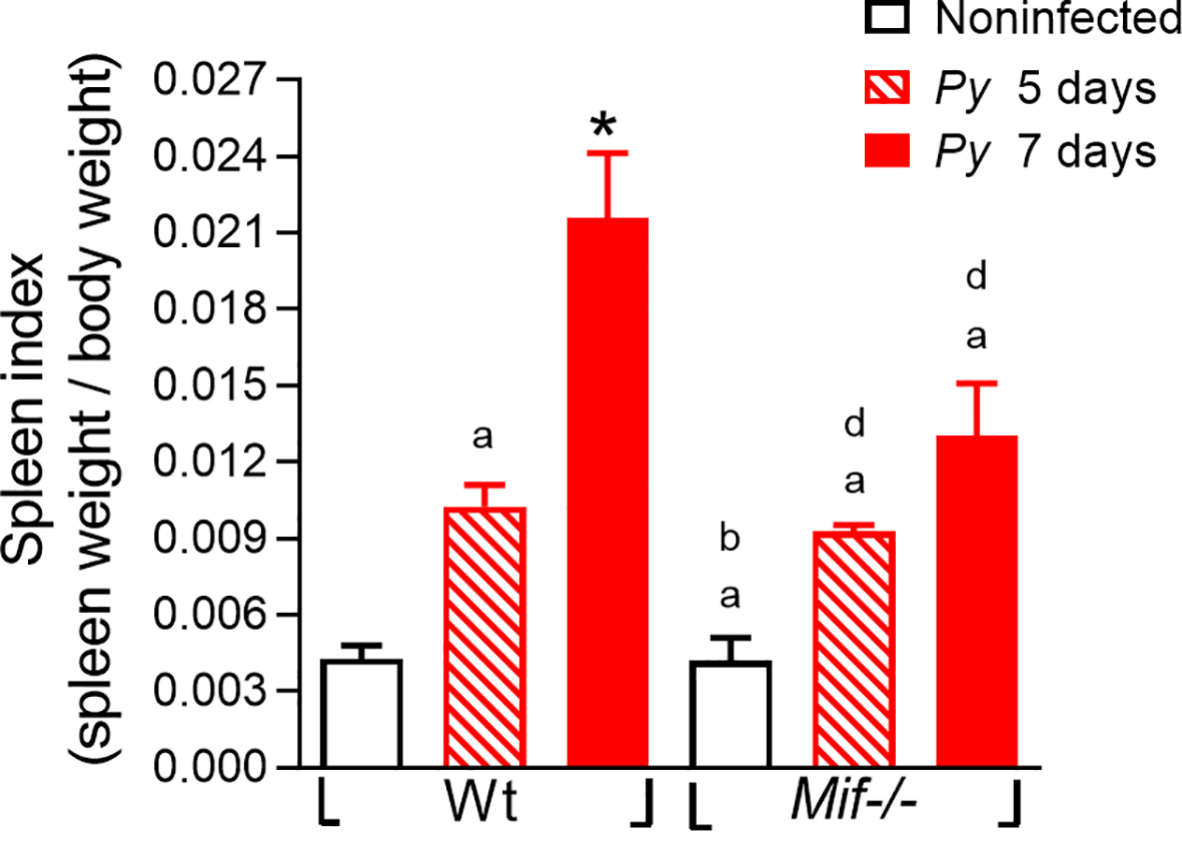
MIF deficiency reduces splenomegaly after *Plasmodium yoelii* 17XL infection. Wild-type and *Mif-/-* mice were infected with 1×10^3^ *Py*17XL-parasitized erythrocytes. Non infected *Mif ^-/-^* and Wt mice were used as controls. The splenic index from infected *Mif ^-/-^* and Wt mice at 5 and 7 days after *Py*17XL infection. Data are expressed as the mean ± SEM and are representative of three independent experiments with at least 3 to 5 mice per group. Values of p < 0.05 were considered statistically significant, (a) compared with the noninfected Wt group, (b) compared with Wt group on day 5th post infection, (*) compared with all experimental groups, and (d) compared with the noninfected *Mif ^-/-^* group.

To assess whether MIF plays a role in the development of splenomegaly in *Plasmodium* infection, the splenic index was determined on days 5 and 7 after *Py*17XL infection. As shown in **Figure 4**, both *Py*17XL-infected *Mif ^-/-^* and Wt groups exhibited a significant increase in spleen size compared to noninfected mice. As shown in **Figure 4**, the *Mif ^-/-^* and Wt groups infected with *Py*17XL exhibited a significant increase in spleen size compared to uninfected mice. At 5 days postinfection, *Mif* ***^-/-^*** mice showed a spleen index similar to *Py*17XL-infected Wt mice. However, at 7 days postinfection, *Mif ^-/-^* mice developed significantly less splenomegaly than Wt mice (**Figure 4**; *, p < 0.0001).

These data demonstrate that MIF promotes splenic damage/splenomegaly caused by *Py*17XL infection.

### *Mif^-/-^* macrophages stimulated with *Py*Ag exhibited higher levels of IL-12 and IL-10 and decreases in TNF-α and nitric oxide production during *Py*17XL infection

The Macrophage-mediated innate immune response controls parasite growth and anemia during acute *Py*17XL infection (Couper et al., 2007), while macrophage proinflammatory secretion is upregulated by MIF (Reyes et al., 2006). To investigate whether *Py*17XL could modify the Mφ response, peritoneal Mφs were isolated 7 days after *Plasmodium* infection and restimulated *in vitro* with *Py*Ag or LPS. Nitric oxide and IL-12, TNF-α, and IL-10 levels were measured.

**Figure 5:**
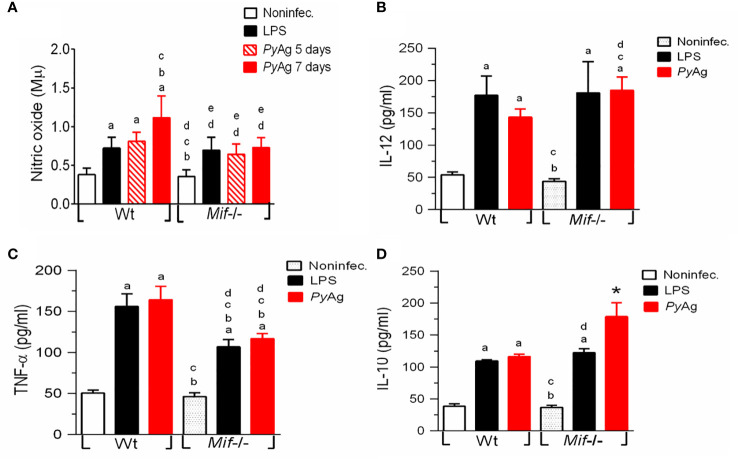
*Mif ^-/-^* macrophages stimulated with *Py*Ag exhibited higher levels of IL-12 and IL-10 and decreases in TNF-α α and nitric oxide production during *Py*17XL infection. Peritoneal Mφs from *Py*17XL-infected Wt and *Mif ^-/-^* experimental groups were isolated at seven days postinfection and stimulated with *Py*Ag (25 µg/mL). **(A)** Nitric oxide, cytokines **(B)** IL-12, **(C)** TNF-α, and **(D)** IL-10 were measured in supernatants collected after 24 h. Data are expressed as the mean ± SEM and are representative of 3 independent experiments with 3- to 5 mice per group. Values of p < 0.05 were considered statistically significant, (a) compared with the noninfected group, (b) compared with the LPS WT group, (c) compared with the *Py*Ag Wt group, (d) compared with the noninfected *Mif ^-/-^* group, and (*) compared with all experimental groups.

*Py*17XL infection promoted higher levels of nitric oxide, IL-12, TNF-α and IL-10 production in both *Mif ^-/-^* and Wt Mφs than in uninfected mice (**Figures 5A–D**. ^a^p < 0.05). However, *Mif ^-/-^* Mφs displayed significantly lower production of nitric oxide and TNF-α at day 7 postinfection than *Py*17XL-infected Wt mice (**Figures 5A, B**). Surprisingly, the magnitude of IL-12 and IL-10 production in Mφs from the *Mif ^-/-^* group was significantly increased compared with *Py*17XL-infected Wt mice (**Figures 5B, D**. ^c^p < 0.05 and ^*^p < 0.0001).

When Mφs from the *Mif ^-/-^* and Wt groups were stimulated *in vitro* with LPS, there were no significant differences in the levels of IL-10 and IL-12 in culture supernatants from *Mif ^-/-^* and Wt mice. In contrast, we found that *Mif ^-/-^* cells produced significantly less TNF-α than *Py*17XL-infected Wt cells (**Figures 5B-D**).

These results show that MIF deficiency reduced the levels of TNF-α and nitric oxide during *Py*17XL infection.

### *Py*17XL infection promotes the proliferative response, high levels of IL-4 and IL-10, and low levels of IFN-γ in *Mif ^-/-^* splenocytes stimulated with *Py*Ag

To determine the role of MIF in the cell proliferation response and cytokine production during *Plasmodium* infection, spleen cells were isolated from *Py*17XL-infected WT and *Mif ^-/-^* mice at 5 and 7 days postinfection. Spleen cells were stimulated with Con-A or *Py*Ag, and the proliferative response was measured by tritiated thymidine (^3^H-TdR) uptake at 72-hours or 5-days, respectively; cytokine production in splenocyte culture supernatants was also measured.

**Figure 6:**
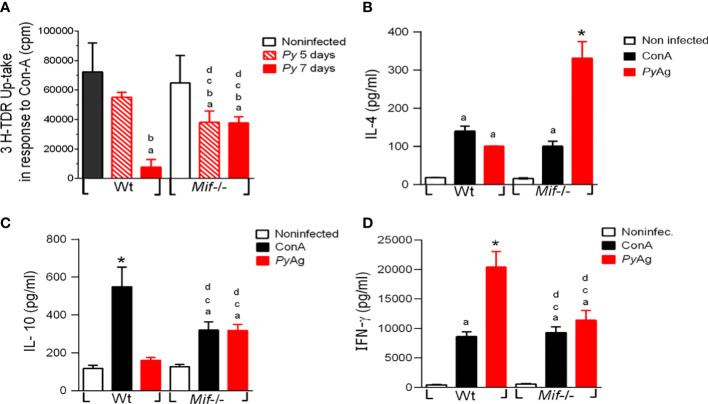
*Plasmodium yoelii* 17XL infection promotes high IL-4 and IL-10 and low IFN-γ production by *Mif ^-/-^* spleen cells after *Py*Ag stimulation. Spleen cells from Wt and *Mif* ^-/-^ mice were isolated at day 7 postinfection. **(A)** Proliferation was assessed in freshly isolated spleen cells upon stimulation with ConA for 5 days. Cytokines **(B)** IL-4, **(C)** IL-10, and **(D)** IFN-γ were measured in supernatants. The bars represent the mean ± SEM incorporation of 3H-TDR from 2 independent experiments. In **(B-D)**, data are expressed as the mean ± SEM and are representative of 3 independent experiments with at least 3 to 5 mice per group. Values of p < 0.05 were considered statistically significant, (a) compared with the noninfected WT group, (b) compared with the ConA+ *Py*-infected Wt group, (c) compared with the *Py*Ag + *Py*-infected Wt group, (d) compared with the noninfected *Mif ^-/-^* group (*) compared with all experimental groups, and (&) compared with the *Py*-infected 5 days post infection Wt group.

*Py*17XL-infected *Mif ^-/-^* splenocytes exhibited a significantly lower proliferative response than Wt mice at 5 days postinfection (**Figure 6A**, ^d^p < 0.0001). However, at 7 days post infection *Mif ^-/-^* mice exhibited higher proliferative responses than Wt mice. In response to *Py*Ag, there were no significant differences in the proliferative response between *Mif ^-/-^* and Wt mice (**Supplementary Figure 1**).

Wild-type splenocytes from infected mice stimulated with *Py*Ag produced significantly higher levels of IL-4 and IFN-γ, and similar levels of IL-10 than splenocytes from noninfected mice (**Figures 6B-D**, respectively). Interestingly, infected *Mif ^-/-^* mice exhibited significantly higher levels of IL-4 and IL-10 (**Figures 6B, C**) and lower levels of IFN-γ than infected Wt mice (**Figure 6D**).

These results showed that MIF deficiency increased the splenocyte proliferative response to Con-A at 7 days postinfection. In response to *Py*Ag MIF deficiency increased levels of IL-4 and IL-10, and decreased levels of IFN-γ were observed.

### MIF deficiency promotes systemic IL-4, IL-10, IL-12, and IL-17 but downregulates IFN-γ production during *Py*17XL infection

To determine whether the decreased susceptibility and pathology of *Mif ^-/-^* mice correlated with the production of systemic cytokines, we compared IL-12, IL-17, TNF-α, IFN-γ, IL-4 and IL-10 levels in sera from *Py*17XL-infected *Mif ^-/-^* and Wt mice at 7 days postinfection.

**Figure 7:**
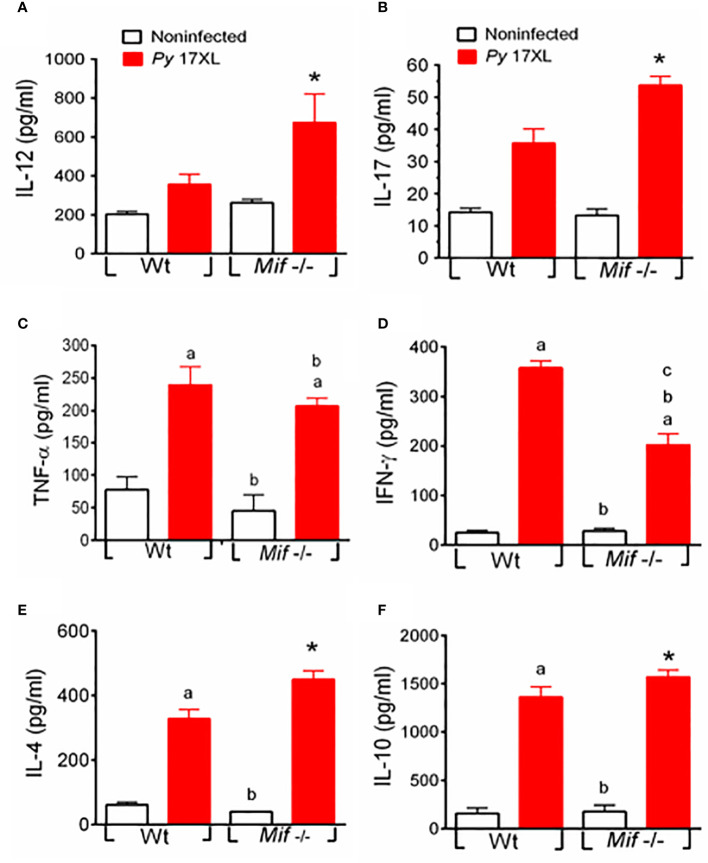
MIF deficiency promotes systemic IL-4, IL-10, IL-12, and IL-17 but downregulates IFN-γ production during *Py*17XL infection. WT and *Mif ^-/-^* mice infected with *Py*17XL were bled 7 days postinfection, and serum samples were obtained. Cytokines **(A)** IL-12, **(B)** IL-17, **(C)** TNF-α, **(D)** IFN-γ, (**E)** IL-4 and **(F)** IL-10 were measured in sera. Data are expressed as the mean ± SEM and are representative of 3 independent experiments with 3 to 5 mice per group. Values of p < 0.05 were considered statistically significant, (a) compared with the noninfected WT group, (b) compared with the *Py*17XL-infected WT group, (c) compared with the noninfected *Mif ^-/-^* group, and (*) compared with all experimental groups.

Both Wt and *Mif ^-/-^* infected mice induced significantly higher levels of IL-12, IL-17, TNF-α, IFN-γ, IL-4, and IL-10 than noninfected mice (**Figures 7A–F**). Notably, *Mif ^-/-^* infected mice produced significantly more IL-12, IL-17, IL-4, and IL-10 than Wt-infected mice (**Figures 7A, B, E, F**, respectively). However, a lower level of IFN-γ was produced compared to Wt. It is important to note that Wt infected mice showed a significant increase in the production of IFN-γ and a decrease in IL-4 compared with *Mif ^-/–^* infected mice at days 5-7 postinfection (**Figure S2C**, and **B**). In addition, IL-10 and IL-12 levels were similar on day 5 postinfection between *Mif ^-/-^* and Wt mice (**Figure S2D**, and **A**).

In summary, *Mif ^-/-^* mice exhibited a mixed Th1/Th2 cytokine profile in serum, as evidenced by elevated levels of IL-12, IL-17, TNF-α, IL-4, and IL-10 and reduced IFN-γ (**Figures 7A-F**).

## Discussion

A large part of the pathogenesis and lethality in *Plasmodium* infection is caused by immune responses that promote proinflammatory-Th1 cytokines (Frimpong et al., 2020). MIF is a cytokine that plays a fundamental role in the inflammatory process and can induce the secretion of proinflammatory factors (Juttner et al., 1998), therefore, MIF could be a determining factor in the induction of a proinflammatory response that directs the course of *Plasmodium* infection, as has been previously suggested (Jaramillo et al., 2004; Griffith et al., 2009).

Here, it was shown that serum MIF levels increased during *Py*17XL infection in Wt mice, and this increase in MIF has also been documented in *Plasmodium*-infected patients (Wang et al., 2009; Han et al., 2010). This finding suggests that MIF may be involved in the pathology. As expected, uninfected *Mif ^-/-^* mice did not have basal levels of MIF in serum; however, a minimal presence of MIF in serum was observed after infection. It is possible that this MIF originates from the parasite, since *Plasmodium* is known to release a MIF homolog (Thorat et al., 2010). In this regard, previous studies in mice, as well as in humans, have shown that *Plasmodium* MIF is present in the host bloodstream (Wang et al., 2009; Han et al., 2010; Miller et al., 2012). Moreover, other intracellular parasites are known to have MIF-like molecules; for example, *Leishmania major* has 2 MIF orthologs that are involved in promoting parasite persistence manipulating the host response by increasing the depletion of protective CD4 T cells (Holowka et al., 2016). Undoubtedly, the role of MIF derived from *Py*17XL in the immune response should be explored in the future.

The murine model of infection with the *Py*17XL strain is lethal to most mouse genetic backgrounds. It is characterized by the development of hyperparasitaemia. In the first 3 days after infection, infected BALB/c mice develop a weak immune response characterized by activation of Mφs, inhibition of dendritic cell (DC) function and maturation, modulation of the activation of regulatory Treg cells (Omer et al., 2003; Abel et al., 2016), low production of IL-6, TNF-α, and IFN-γ, absence of IL-4 and IL-12 and increased levels of TGF-β and IL-10 (Chen et al., 2010; Fu et al., 2012a). However, 5 days after infection, an inflammatory response associated with overexpression of IFN-γ and TNF-α in serum, and PD-1 expression on CD4+ T cells are significantly increased (Salazar-Castanon et al., 2018; Wang et al., 2021). The mice exhibited weight loss, and developed splenomegaly, anemia, and death approximately 7 to 11 days after infection (Chen et al., 2010; Fu et al., 2012b).

Here, survival was significantly improved in *Py*17XL-infected *Mif ^-/-^* mice; 11 days postinfection all Wt mice succumbed, while more than 50% of *Mif ^-/-^* mice survived. This higher survival rate was associated with significantly reduced parasitemia in the first 7 days post-infection. It is important to note that the Py17XL parasite continued to replicate 11 days postinfection in surviving *Mif ^-/-^* mice, which achieved very high parasitemia. However, mice survived 10 days longer than Wt mice, indicating that survival of *Mif ^-/-^* mice was independent of parasite load. These results differ from other reports that show higher mortality in *Plasmodium* infection according to parasitemia density (Lamb et al., 2006), but our results are consistent with the report that showed that mortality is independent of parasite load in *Plasmodium chabaudi* (Lamb and Langhorne, 2008). These discrepancies may be due to differences in the virulence of the different *Plasmodium* strains used.

Although the inflammatory immune response controls parasite replication (Chen et al., 2014), excessive or dysregulated inflammatory response promotes cachexia (Smith et al., 2007; Remels et al., 2013), anemia (Lamikanra et al., 2009; Thawani et al., 2014), splenomegaly and death (McGregor et al., 2015). Interestingly, infected *Mif ^-/-^* mice exhibited lower splenic index, delayed development of cachexia, and reduced early hemoglobin loss compared to infected Wt mice that could be associated with a reduced inflammatory response. In further support of this observation, administration of the mAb anti-TNF-α (in combination with anti-IFN-γ, or not) reduced splenomegaly in mice infected with *P. chabaudi* (Jacobs et al., 1996). Moreover, MIF has been shown to induce a catabolic effect in muscle and anti-MIF mAb to increase muscle in TNFα *^-/-^* mice (Benigni et al., 2000). Previous reports have shown that *Mif ^-/-^* mice infected with *P. chabaudi* developed less severe anemia and a higher survival rate associated with suppression of erythropoiesis by MIF (Martiney et al., 2000; McDevitt et al., 2006; Malu et al., 2011).

The partial protection observed in the infected *Mif ^-/-^* mice could be associated with the reduction of the Th1-inflammatory profile of these mice. We observed a strong relationship between these cytokine levels and a lower incidence of pathology and higher survival in the *Mif ^-/-^* group. Specifically, *Py*Ag-stimulated peritoneal Mφs showed a reduction in the proinflammatory cytokines TNF-α and NO and an increase in the anti-inflammatory cytokine IL-10. In addition, spleen cells showed a significant increase in the proliferative response to Con-A, low levels of the Th1 cytokine IFN-γ, and high levels of the anti-inflammatory cytokines-Th2, IL-4 and IL-10. These results are consistent with the finding that *Mif ^-/-^* CD4+ T cells have an increased ability to proliferate (Malu et al., 2011), as MIF neutralization markedly increases the proliferation of splenic T cells (Abe et al., 2001). Additionally, IFN-γ-deficient, and IFN-γ-depleted mice had less cachexia, a reduced splenic index, and a longer survival time than *Py* N67C-infected Wt mice (Lacerda-Queiroz et al., 2017).

Although the proinflammatory cytokines, IL-12, IFN-γ, and TNF-α promote resistance and providing protection against *Plasmodium* infection (Stevenson et al., 1995; Mohan et al., 1997), high levels of IFN-γ and TNF-α in serum have also been associated with severe anemia (Yap and Stevenson, 1994; May et al., 2000), cachexia (Cannon et al., 2007; Remels et al., 2010) and pathology of malaria disease (Hunt and Grau, 2003; Vogetseder et al., 2004; Prakash et al., 2006). Thus, a balance between inflammatory and anti-inflammatory cytokines is essential for parasitemia control and host survival.

Our results demonstrated that infected *Mif ^-/-^* mice exhibited high levels of IL-4, IL-10, IL-12, and IL-17 in serum, but low levels of IFN-γ than infected Wt mice. Various reports have shown that IFN-γ is associated with pathology in *Plasmodium* infection. For example, low levels of IFN-γ and increased serum levels of IL-4 and IL-10 prevented severe pathology in mice infected with the nonlethal strain *Py*17XNL (Couper et al., 2008; Bakir et al., 2011). Similarly, IFN-γ deficient and IFN-γ-depleted mice had higher body weights, reduced splenic damage, and longer survival times than *Py*N67C-infected WT mice (Lacerda-Queiroz et al., 2017). In contrast, increased resistance to *P. chabaudi adami* infection in *Mif ^-/-^* mice was associated with increased IFN-γ production and reduced IL-4 and IL-10 in early infection (Malu et al., 2011). These contrasting data on the role of IFN-γ can be explained by the difference in the *Plasmodium* strain used, and it is likely that high levels of IFN-γ are required for the control of nonlethal strains (Langhorne et al., 2004), while in lethal strains of *Plasmodium*, high IFN-γ seems to be associated to mortality or pathology. Our results show that infected *Mif ^-/-^* mice exhibited lower amounts of IFN-γ than Wt mice, although their low IFN-γ levels were maintained throughout the infection were sufficient to partially reduce parasitemia in early stages, reduce pathology and prolong survival in *Py*17XL infection.

Furthermore, the reduced pathology in *Mif ^-/–^* infected mice may be associated with increased IL-10 both in serum and of Mφs supernatant, together with decreased IFN-γ in serum and spleen cell supernatant, and low levels of TNF-α and NO in the supernatant. This is supported by the observation that low levels of IL-10 together with high levels of TNF-α have been associated with anemia and cachexia (Kurtzhals et al., 1998; May et al., 2000). IL-10 knockout mice exhibited severe pathology and death (Sanni et al., 2004). In addition, the administration of anti-IFN-γ or anti-TNF-α mAb reduces pathology and mortality (Li et al., 1999; Sanni et al., 2004). It is possible that the decrease in TNF-α and NO production by peritoneal Mφs limited the progression of serious life-threatening complications in the *Mif ^-/-^* group. Although a significant reduction in serum TNF-α was not observed in infected *Mif ^-/-^* mice compared to Wt mice, the significant increase in serum IL-10 and IL-4 could negatively regulate the inflammation causing the pathology. In fact, a therapeutic strategy against cachexia is the administration of anti-inflammatory cytokines (Argiles et al., 2011).

Finally, the increased survival observed in *Mif ^-/-^* mice could be explained, at least in part, by delay and reduction of anemia, since the anemia generated by *Py*17XL infection induces early mortality (Chen et al., 2014). Here, the reduced anemia could be associated with the high serum levels of IL-10, and IL-12 and the deficiency of TNF-α, IFN-γ and MIF. Malaria anemia depends on a fine mechanism of suppression of hematopoiesis by a particular combination of some inflammatory cytokines. For example, low levels of IL-12 and high levels of MIF have been shown to be associated with severe malaria (Chaiyaroj et al., 2004; Langhorne et al., 2004). Administration of recombinant IL-12 (rIL-12) confers protection against parasitemia, severe anemia, and mortality in *P. chabaudi* infection (Mohan and Stevenson, 1998; Su and Stevenson, 2002). Furthermore, during nonlethal infection with *Py*17XNL, anemia was also associated with reduced levels of IL-17 (Xu et al., 2013). The administration of rIL-17, and NO blockade, promote erythropoiesis by stimulating the development of early growth of erythroid progenitors (Krstic et al., 2010; Krstic et al., 2012).

## Conclusion

The data showed here demonstrate that *Mif ^-/-^* mice infected with the *Py*17XL had an improved survival rate, reduced cachexia, delayed anemia, and prevented splenomegaly. This was associated with increased levels of IL-4, IL-10, IL-12, and IL-17 in serum; IL-10 in the supernatant of macrophages and IL-4 and IL-10 in the supernatant from spleen cells. The reduced levels of IFN-γ in serum and spleen cells, as well as the decrease in TNF-α and NO by macrophages.

Together, these data indicate that MIF has an important role as a mediator of cytokines involved in the immune response associated with pathogenesis and host lethality in *Plasmodium* infection. It is important to take these observations into account to prevent and reduce complications in *Plasmodium* infections.

## Data availability statement

The original contributions presented in the study are included in the article/**Supplementary Material**. Further inquiries can be directed to the corresponding authors.

## Ethics statement

All procedures performed in this work were in accordance with the ethical standards approved and carried out under strict accordance with the guidelines for the Care and Use of Laboratory Animals adopted by the U.S. National Institutes of Health, and the Mexican Regulation of Animal Care and maintenance (NOM-062-ZOO-1999, 2001). The study was revised and approved by the Ethics Committee at FES-Iztacala, UNAM (CE/FESI/032017/1154).

## Author contributions

All the authors significantly contributed to this work. VS-C, ML-H, and MR-S designed the study; VS-C and IJ-A conducted the study selection and data extraction; VS-C and MR-S performed the data analysis and genetic model assessment and prepared the figures; VS-C, ML-H, and MR-S drafted the manuscript; all authors revised it critically for important intellectual content and approved the final draft.

## Funding

This work was partially supported by the following projects from the Programa de Apoyo a Proyectos de Investigación e Innovación Tecnológica (PAPIIT), UNAM, grant number IN228620 to ML-H and IN217021 to MR-S.

## Acknowledgments

VHS-C is the recipient of a scholarship from the Programa de Becas Posdoctorales, DGAPA-UNAM, Mexico.

## Conflict of interest

The authors declare that the research was conducted in the absence of any commercial or financial relationships that could be construed as a potential conflict of interest.

## Publisher’s note

All claims expressed in this article are solely those of the authors and do not necessarily represent those of their affiliated organizations, or those of the publisher, the editors and the reviewers. Any product that may be evaluated in this article, or claim that may be made by its manufacturer, is not guaranteed or endorsed by the publisher.

## Supplementary material

Supplementary material S1 No significant differences in the proliferative response *Py*Ag between *Mif ^-/-^* and Wt mice. Seven days post-*Py* 17XL infection, the spleen cell proliferation was assayed in freshly isolated spleen cells upon stimulation with *Py*Ag for 5 days. The bars represent the mean incorporation of ^3^H-TDR. Data are expressed as the mean ± SEM and are representative of 2 independent experiments with 3 to 5 mice per group.

Supplementary material S2 Wild-type infected mice promotes increase of IFN-γ but decreased of IL-4 production during *Py*17XL infection. WT and *Mif ^-/-^* mice infected with *Py*17XL were bled 5 and 7 days postinfection, and serum samples were obtained. Cytokines **(A)** IL-12, **(B)** IFN-γ, **(C)** IL-4, and **(D)** IL-10 were measured in sera. Data are expressed as the mean ± SEM and are representative of 3 independent experiments with 3 to 5 mice per group. Values of p < 0.05 were considered statistically significant, (a) compared with the noninfected WT group, (b) compared with the *Py*17XL -infected WT group, (c) compared with the noninfected *Mif ^-/-^* group, and (*) compared with all experimental groups.

Supplementary material S3 Representative curves for ELISA cytokines: MIF, IL-4, IL-10, IL-12, IL-17, IFN-γ, and TNF-α for OD. conversion.
