# Erratum: Macrophage migration inhibitor factor contributes to immunopathogenesis during *Plasmodium yoelii* 17XL infection

**DOI:** 10.3389/fcimb.2022.1106479

**Published:** 2022-12-15

**Authors:** Frontiers Production Office

**Affiliations:** Frontiers Media SA, Lausanne, Switzerland

**Keywords:** P. yoelii 17XL, host-pathogen interaction, parasite, MIF, malaria, proinflammatory cytokines, pathogenesis, immune response

Due to a production error, there was an error in the affiliations. Instead of:

“Víctor H. Salazar-Castañón^1^, Imelda Juárez-Avelar^1^, Martha Legorreta-Herrera^2^* and Miriam Rodriguez-Sosa^1^*

^1^Laboratorio de Inmunidad Innata, Unidad de Investigación en Biomedicina, Facultad de Estudios Superiores Iztacala, UNAM, Estado de México, México, ^2^Laboratorio de Inmunología Molecular, Unidad de Investigación Química Computacional, Síntesis y Farmacología en Moléculas de Interés Biológico, División de Estudios de Posgrado e Investigación, Facultad de Estudios Superiores Zaragoza, Universidad Nacional Autónoma de México (UNAM), Ciudad de México, México.”

It should be:

“Víctor H. Salazar-Castañón^1^, Imelda Juárez-Avelar^2^, Martha Legorreta-Herrera^1^* and Miriam Rodriguez-Sosa^2^*

^1^Laboratorio de Inmunología Molecular, Unidad de Investigación Química Computacional, Síntesis y Farmacología en Moléculas de Interés Biológico, División de Estudios de Posgrado e Investigación, Facultad de Estudios Superiores Zaragoza, Universidad Nacional Autónoma de México (UNAM), Ciudad de México, México. ^2^Laboratorio de Inmunidad Innata, Unidad de Investigación en Biomedicina, Facultad de Estudios Superiores Iztacala, UNAM, Estado de México, México”

The publisher apologizes for this mistake.

The original version of this article has been updated.

