# Supplementary figures and images for: Macrophage migration inhibitory factor contributes to immunopathogenesis during Plasmodium yoelii 17XL infection
Source: Front Cell Infect Microbiol. 2022 Aug 24;12:968422. doi: 10.3389/fcimb.2022.968422 (PMC9449124; doi:10.3389/fcimb.2022.968422)

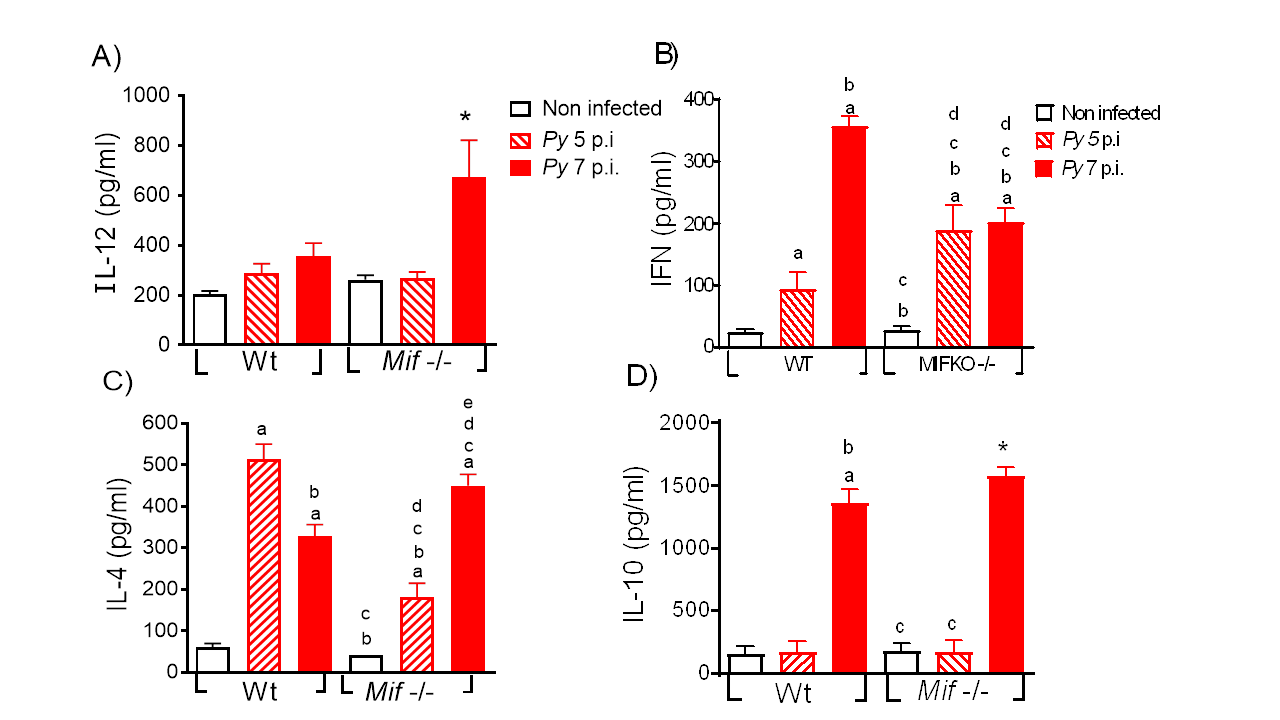

Supplement: Supplementary material S2 — Wild-type infected mice promotes increase of IFN-γ but decreased of IL-4 production during Py17XL infection. WT and Mif -/- mice infected with Py17XL were bled 5 and 7 days postinfection, and serum samples were obtained. Cytokines (A) IL-12, (B) IFN-γ, (C) IL-4, and (D) IL-10 were measured in sera. Data are expressed as the mean ± SEM and are representative of 3 independent experiments with 3 to 5 mice per group. Values of p < 0.05 were considered statistically significant, (a) compared with the noninfected WT group, (b) compared with the Py17XL -infected WT group, (c) compared with the noninfected Mif -/- group, and (*) compared with all experimental groups. [file Image_2.tif]
