# Supplementary material for: Macrophage migration inhibitory factor contributes to immunopathogenesis during Plasmodium yoelii 17XL infection
Source: Front Cell Infect Microbiol. 2022 Aug 24;12:968422. doi: 10.3389/fcimb.2022.968422 (PMC9449124; doi:10.3389/fcimb.2022.968422)

Fig. S3. A representative curve for each Cytokine ELISA for converting OD

Representative Curve of MIF

	1	2	3	4	5	6	7	8	9	10	11	12
A	0.278	0.215	0.188	0.164	0.128	0.135	0.134	0.107	0.149	0.107	0.151	0.15
B	0.279	0.138	0.137	0.103	0.114	0.109	0.096	0.118	0.099	0.121	0.096	0.127
C	0.211	0.128	0.104	0.109	0.129	0.107	0.076	0.091	0.09	0.137	0.109	0.092
D	0.181	0.113	0.16	0.117	0.097	0.081	0.08	0.091	0.11	0.097	0.08	0.08
E	0.183	0.144	0.16	0.105	0.114	0.091	0.087	0.081	0.108	0.123	0.123	0.105
F	0.173	0.113	0.081	0.082	0.125	0.079	0.137	0.078	0.111	0.083	0.15	0.096
G	0.151	0.097	0.109	0.082	0.119	0.086	0.11	0.095	0.165	0.122	0.143	0.11
H	0.142	0.1	0.092	0.099	0.095	0.091	0.117	0.088	0.136	0.112	0.15	0.117

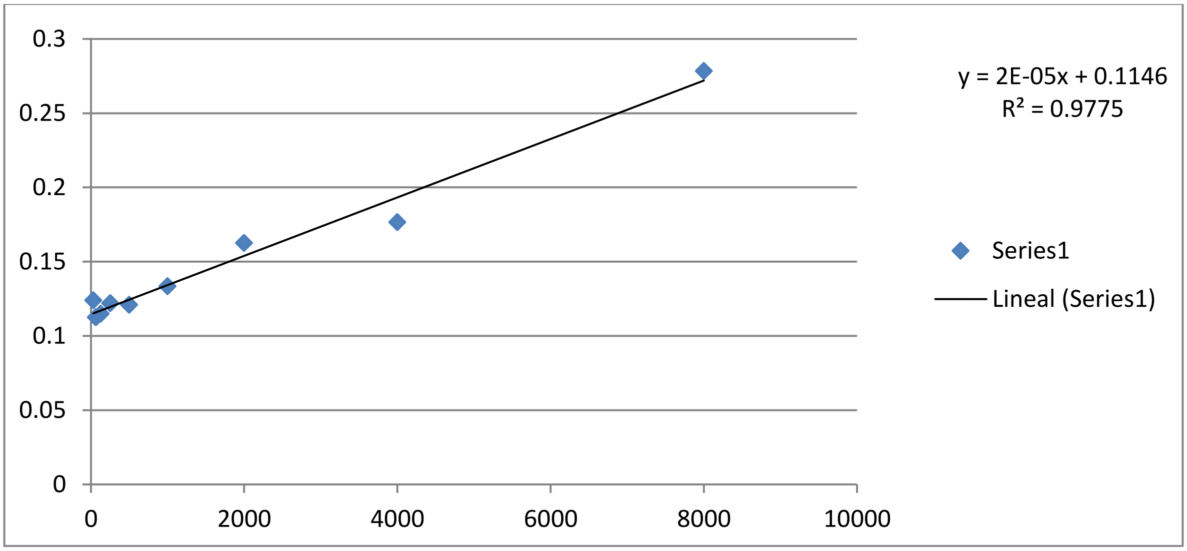

Representative Curve of IL-4

	1	2	3	4	5	6	7	8	9	10	11	12	
A	0.673	0.567	0.589	0.527	0.46	0.42	0.336	0.308	0.24	0.155	0.157	0.074	405
B	0.563	0.5	0.517	0.429	0.414	0.36	0.3	0.268	0.24	0.159	0.149	0.063	405
C	0.246	0.257	0.231	0.231	0.226	0.24	0.242	0.247	0.24	0.243	0.269	0.251	405
D	0.252	0.237	0.262	0.254	0.26	0.26	0.288	0.268	0.27	0.263	0.267	0.278	405
E	0.334	0.233	0.262	0.253	0.252	0.29	0.281	0.26	0.25	0.264	0.255	0.276	405
F	0.266	0.24	0.253	0.239	0.248	0.26	0.26	0.245	0.3	0.264	0.267	0.28	405
G	0.252	0.268	0.253	0.236	0.266	0.26	0.271	0.249	0.24	0.223	0.277	0.264	405
H	0.232	0.136	0.298	0.264	0.257	0.25	0.244	0.228	0.23	0.247	0.283	0.251	405

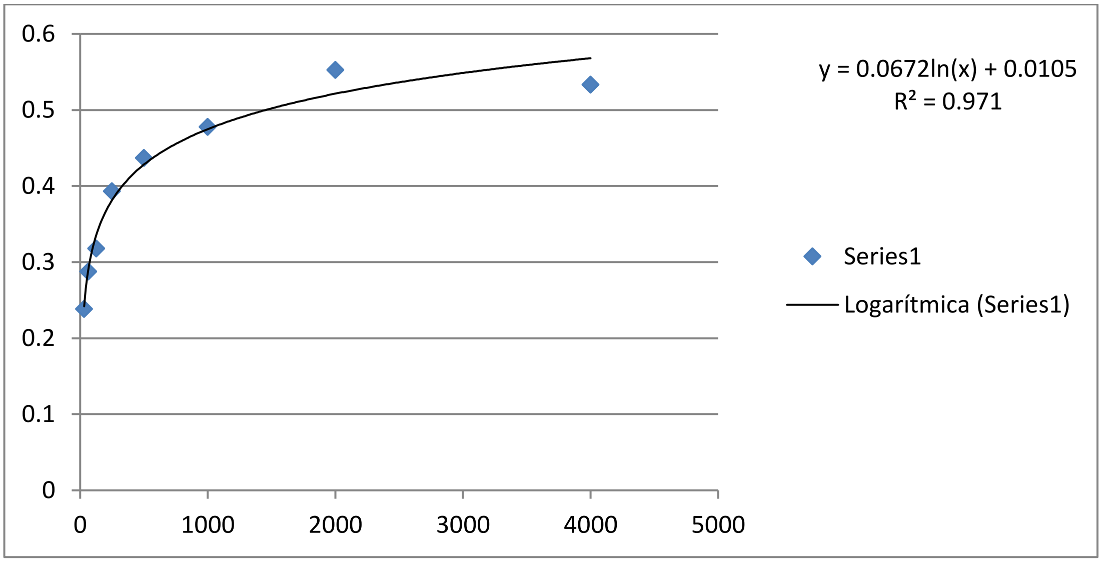

Representative Curve of IL-10

	1	2	3	4	5	6	7	8	9	10	11	12	
A	1.118	0.973	0.846	0.879	0.683	0.618	0.559	0.438	0.377	0.224	0.129	0.07	405
B	1.076	1.062	0.892	0.842	0.84	0.604	0.554	0.434	0.351	0.249	0.149	0.06	405
C	0.306	0.327	0.453	0.435	0.404	0.332	0.391	0.332	0.353	0.348	0.307	0.33	405
D	0.301	0.325	0.444	0.367	0.364	0.378	0.336	0.34	0.398	0.355	0.305	0.3	405
E	0.309	0.334	0.362	0.32	0.327	0.328	0.375	0.375	0.349	0.347	0.361	0.34	405
F	0.311	0.347	0.333	0.328	0.351	0.322	0.379	0.333	0.325	0.342	0.333	0.33	405
G	0.348	0.344	0.309	0.31	0.349	0.331	0.351	0.324	0.34	0.333	0.32	0.29	405
H	0.335	0.432	0.322	0.304	0.336	0.311	0.31	0.31	0.32	0.306	0.289	0.28	405

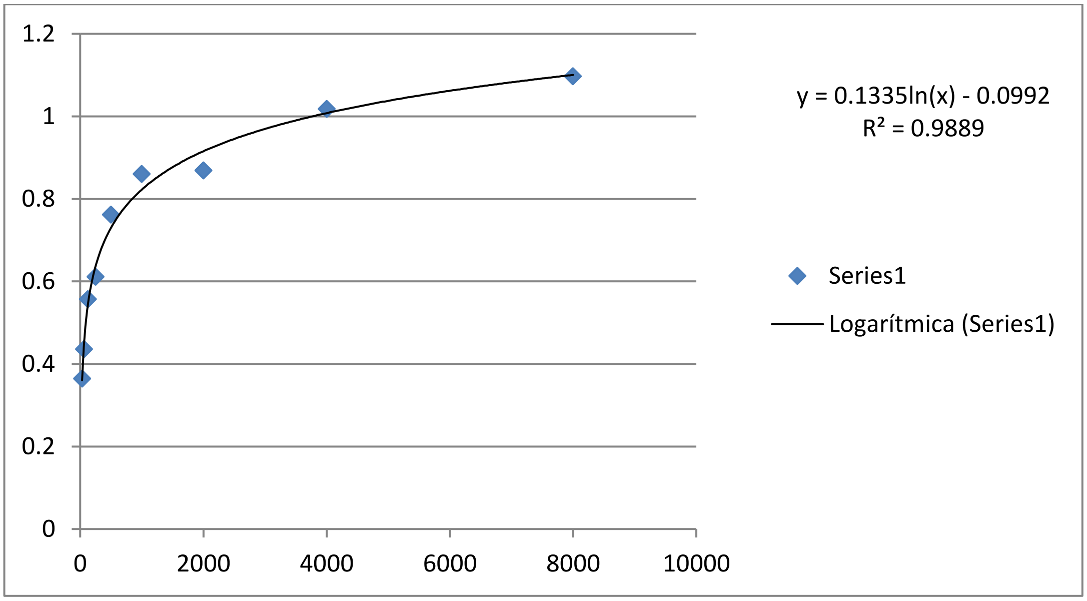

Representative Curve of IL-12

	1	2	3	4	5	6	7	8	9	10	11	12	
A	0.451	0.301	0.281	0.267	0.199	0.149	0.112	0.093	0.107	0.065	0.059	0.061	405
B	0.411	0.301	0.307	0.27	0.234	0.203	0.148	0.121	0.102	0.084	0.07	0.063	405
C	0.107	0.101	0.111	0.108	0.096	0.108	0.088	0.098	0.091	0.082	0.069	0.066	405
D	0.102	0.113	0.118	0.118	0.109	0.121	0.086	0.076	0.071	0.078	0.071	0.062	405
E	0.087	0.091	0.101	0.1	0.102	0.103	0.084	0.111	0.086	0.082	0.073	0.068	405
F	0.096	0.099	0.112	0.1	0.099	0.102	0.086	0.105	0.085	0.081	0.083	0.089	405
G	0.118	0.104	0.109	0.12	0.066	0.075	0.097	0.093	0.089	0.082	0.072	0.073	405
H	0.119	0.126	0.103	0.1	0.086	0.069	0.085	0.078	0.083	0.114	0.094	0.091	405

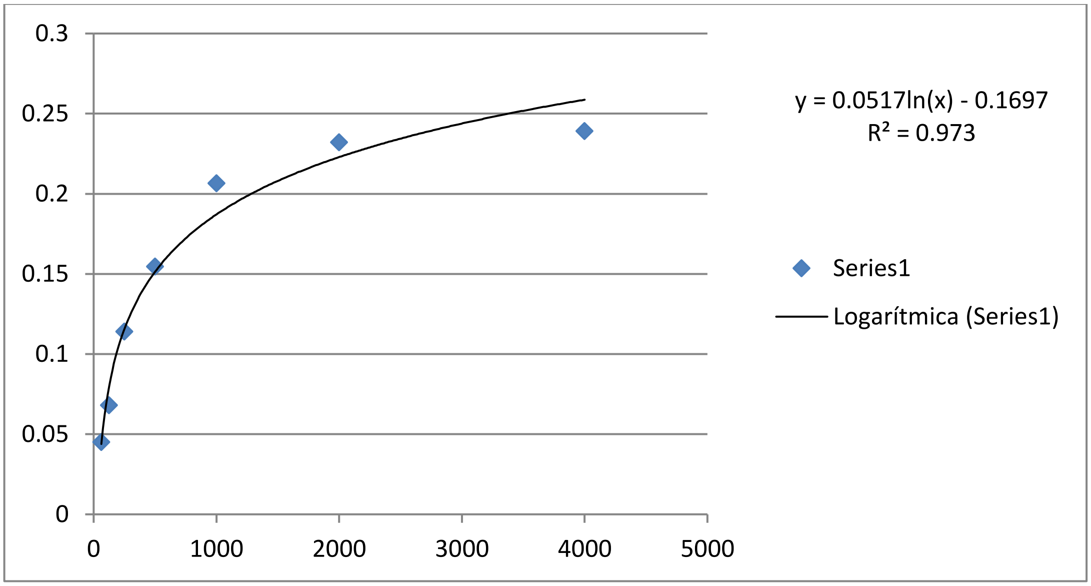

Representative Curve of IL-17

	1	2	3	4	5	6	7	8	9	10	11	12	405
A	1.152	1.01	0.871	0.858	0.721	0.703	0.66	0.568	0.492	0.3	0.251	0.071	405
B	1.219	0.976	0.944	0.863	0.726	0.75	0.583	0.576	0.514	0.372	0.235	0.08	405
C	0.431	0.566	0.431	0.438	0.489	0.428	0.472	0.439	0.443	0.434	0.43	0.461	405
D	0.424	0.578	0.438	0.432	0.525	0.428	0.424	0.437	0.443	0.414	0.433	0.451	405
E	0.497	0.414	0.48	0.447	0.565	0.418	0.44	0.504	0.404	0.433	0.444	0.42	405
F	0.521	0.429	0.448	0.426	0.48	0.444	0.463	0.426	0.419	0.506	0.444	0.453	405
G	0.437	0.539	0.411	0.42	0.547	0.416	0.445	0.428	0.473	0.445	0.469	0.482	405
H	0.467	0.523	0.454	0.442	0.518	0.42	0.435	0.42	0.476	0.412	0.44	0.403	405

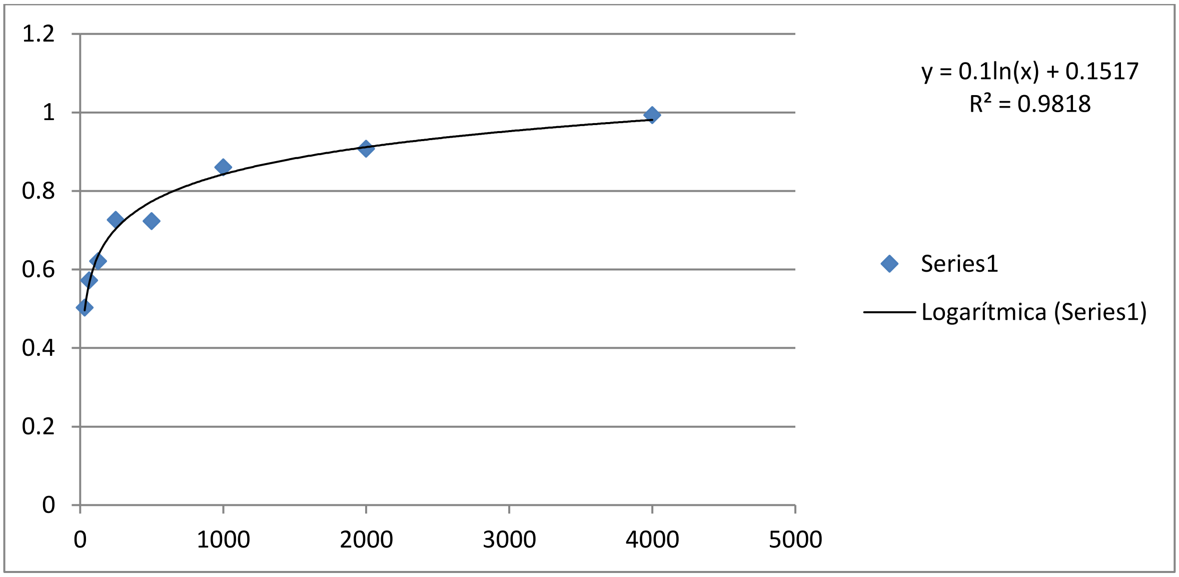

Representative Curve of TNF-α

	1	2	3	4	5	6	7	8	9	10	11	12	
A	0.466	0.364	0.348	0.315	0.232	0.161	0.117	0.108	0.087	0.076	0.081	0.084	405
B	0.499	0.434	0.363	0.321	0.269	0.181	0.139	0.111	0.087	0.09	0.072	0.086	405
C	0.086	0.082	0.1	0.108	0.093	0.09	0.092	0.09	0.083	0.079	0.079	0.078	405
D	0.094	0.089	0.09	0.086	0.092	0.091	0.089	0.088	0.131	0.079	0.083	0.076	405
E	0.077	0.078	0.105	0.087	0.092	0.09	0.083	0.089	0.087	0.083	0.08	0.074	405
F	0.079	0.085	0.082	0.088	0.097	0.102	0.081	0.087	0.084	0.078	0.077	0.073	405
G	0.08	0.081	0.085	0.091	0.082	0.078	0.078	0.08	0.083	0.082	0.075	0.07	405
H	0.075	0.079	0.086	0.084	0.075	0.073	0.082	0.076	0.08	0.084	0.074	0.068	405

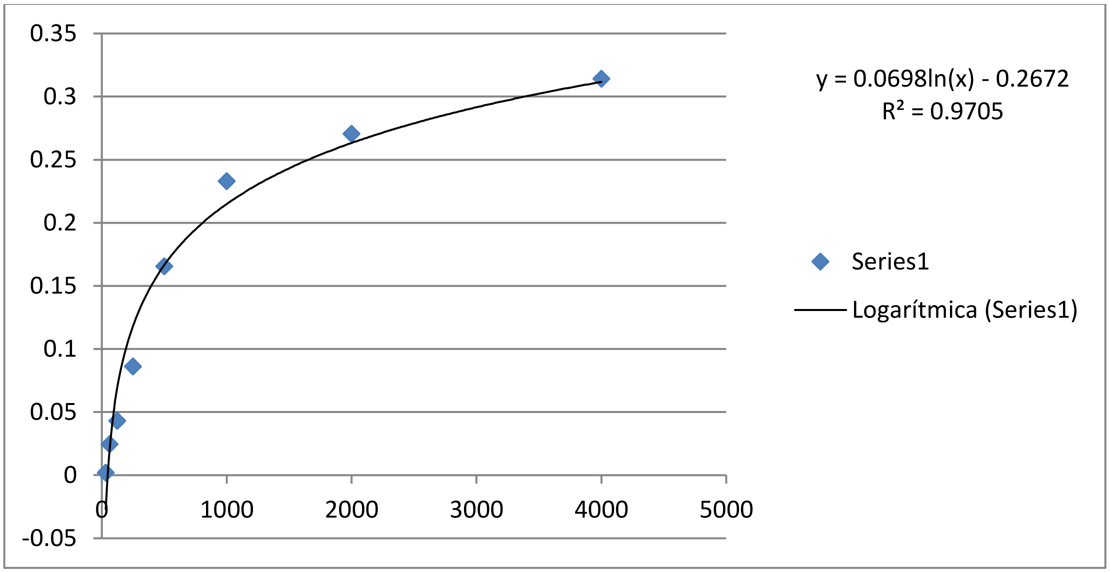

Representative Curve of IFN-γ

	1	2	3	4	5	6	7	8	9	10	11	12	
A	0.924	0.697	0.601	0.527	0.499	0.45	0.401	0.332	0.257	0.257	0.129	0.069	405
B	0.96	0.837	0.66	0.584	0.531	0.376	0.351	0.304	0.302	0.281	0.18	0.073	405
C	0.236	0.236	0.316	0.3	0.297	0.265	0.285	0.255	0.248	0.248	0.224	0.232	405
D	0.209	0.245	0.344	0.273	0.286	0.281	0.249	0.256	0.286	0.272	0.229	0.224	405
E	0.244	0.243	0.259	0.248	0.244	0.225	0.257	0.278	0.286	0.278	0.277	0.233	405
F	0.241	0.233	0.274	0.226	0.282	0.271	0.271	0.263	0.252	0.254	0.26	0.25	405
G	0.251	0.255	0.229	0.237	0.263	0.235	0.256	0.235	0.266	0.239	0.224	0.212	405
H	0.238	0.36	0.221	0.233	0.259	0.241	0.23	0.239	0.224	0.245	0.213	0.209	405

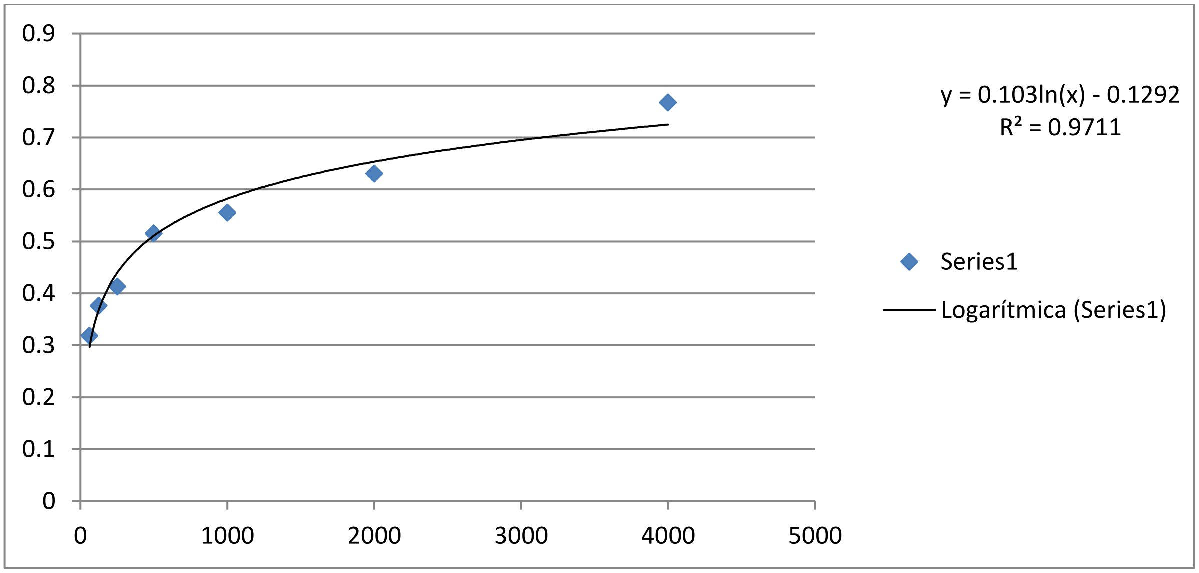

Supplement: Supplementary material S3 — Representative curves for ELISA cytokines: MIF, IL-4, IL-10, IL-12, IL-17, IFN-γ, and TNF-α for OD. conversion. [file DataSheet_1.pdf]
